# Improvement of molecular-replacement models with *Sculptor*

**DOI:** 10.1107/S0907444910051218

**Published:** 2011-03-18

**Authors:** Gábor Bunkóczi, Randy J. Read

**Affiliations:** a CIMR Haematology, University of Cambridge, Wellcome Trust/MRC Building, Hills Road, Cambridge CB2 0XY, England

**Keywords:** molecular replacement, model improvement, residue-substitution score

## Abstract

In molecular replacement, the quality of models can be improved by transferring information contained in sequence alignment to the template structure. A family of algorithms has been developed that make use of the sequence-similarity score calculated from residue-substitution scores smoothed over nearby residues to delete or downweight parts of the model that are unreliable. These algorithms have been implemented in the program *Sculptor*, together with well established methods that are in common use for model improvement. An analysis of the new algorithms has been performed by studying the effect of algorithm parameters on the quality of models. Benchmarking against existing tech­niques shows that models from *Sculptor* compare favourably, especially if the alignment is unreliable. Carrying out multiple trials using alternative models created from the same structure but using different algorithm parameters can significantly improve the success rate.

## Abstract 2

The molecular-replacement model-improvement program *Sculptor* is described, with an analysis of the algorithms used.

## 1. Introduction   

Molecular replacement may prove difficult for several reasons: the data quality may be low, the structure may suffer from translational pseudosymmetry or other pathologies, the model may be severely incomplete or (more typically) a very distant homologue. Although some of these difficulties only apply in certain special cases, preparing the best model for a rigid unit (be it a monomer in a multimeric protein, a domain in a multidomain protein or the whole macromolecule in the case of single-domain single-copy structures) is of basic importance in all molecular-replacement searches. Usually structures of homologous proteins are used for this purpose, although *ab initio* models have also proved successful (Qian *et al.*, 2007 ▶; Rigden *et al.*, 2008 ▶). However, the unedited structure of a related protein is not necessarily the best model for the target structure and it is common practice to perform modifications to improve the similarity. Homology modelling is a very powerful technique to make improvements (Qian *et al.*, 2007 ▶) but is computationally intensive, and for certain applications there is a need for simple quick algorithms.

Although many sensible procedures had been in circulation for a long time, systematic investigations on the topic started with the seminal paper of Schwarzenbacher *et al.* (2004 ▶). Based on experience with a large set of structures, they formulated several simple modifications that were found to lead to improvements in the majority of cases. These algorithms have since been implemented in the *CCP*4 (Winn *et al.*, 2011 ▶) programs *CHAINSAW* (Stein, 2008 ▶) and *MOLREP* (Vagin & Teplyakov, 2010 ▶). Additional possibilities for improvement were subsequently described by Lebedev *et al.* (2008 ▶) and implemented in *MOLREP*. A common property of these methods is that they use information present in the sequence alignment and also in the model structure itself to make simple modifications.

However, the available information is not yet optimally exploited with current methods and making use of further information could potentially improve the rate of success for molecular replacement. In this paper, we describe novel algorithms that allow the capture and combination of information from different sources and investigate their effects on molecular-replacement models.

## 2. Procedures   

*Sculptor* offers three modification options, which can be run in any combination: main-chain deletion, side-chain pruning and *B*-factor modification. *Sculptor* takes a model structure and optional alignments to perform its tasks. A procedure has been developed to distinguish protein chains from all others (termed hetero chains) in order to allow the input of model files containing ligands and solvent. Firstly, the chain type is established based on the residue content. Protein chains are identified by most residues being common amino acids, but a small number of unknown residues (typically modified residues such as selenomethionine and oxidized cysteine) are allowed. Incidentally, this also helps to minimize structural gaps in the processed model, since modified residues can be included as long as their backbone atoms are named consistently. This procedure may therefore be more robust than making a decision based on a record being ATOM or HETATM and also leads to slightly higher model completeness. Protein chains are then paired with a corresponding alignment (or none if only requesting modifications that do not require alignment information) and undergo the requested modification steps. Hetero chains are processed by deleting everything except specifically named compounds, *e.g.* a haem can be retained for haem-binding proteins.

### 2.1. Sequence-similarity calculation   

As the available scoring matrices are on different scales, normalization is performed so that a perfectly matching alignment would give a score around 1.0, a randomly aligned sequence (including random matches) a score around 0.0 and segments containing consecutive residues that align with gap positions a score around −1.0. These normalized matrices are used in all subsequent steps. *Sculptor* currently includes the identity, PAM250 (Dayhoff *et al.*, 1978 ▶), BLOSUM50 and BLOSUM62 (Henikoff & Henikoff, 1992 ▶) matrices.

**Figure 1:**
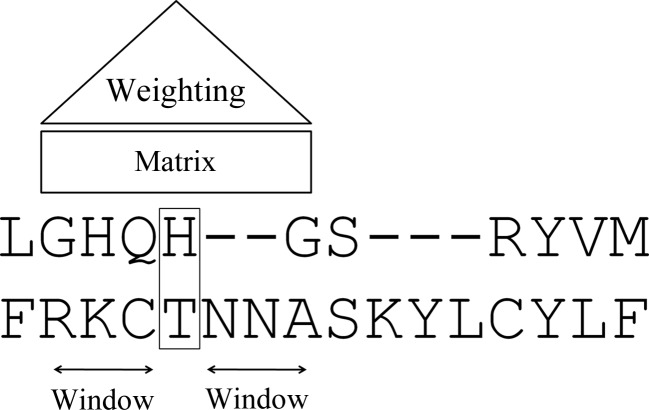
Graphical representation of the parameters used in sequence-similarity calculations.

To calculate sequence-similarity scores, each alignment position is scored with the selected matrix. Subsequently, these primary scores are combined into a moving average by taking into account a certain number of alignment positions (averaging window) on either side of the position in question with a simple triangular weighting scheme that gives maximum weight to the central position, linearly decreasing with the distance of the position from the centre (Fig. 1 ▶).

### 2.2. Accessible surface-area calculation   

*Sculptor* implements a simple algorithm described by Shrake & Rupley (1973 ▶) in which the surface of each atom is approximated by a spherical mesh and each point in the mesh is checked for overlap with every other atom in the structure. Implementation is based on the discussion at http://boscoh.com/protein/calculating-the-solvent-accessible-surface-area-asa.

### 2.3. Main-chain deletion   

The decision on whether a residue should be kept or deleted from the model is made based on the sequence-similarity score that corresponds to the given alignment position. In addition to the parameters of the sequence-similarity calculation, this requires a suitably chosen threshold value above which the residue is kept and below which it is discarded. This algorithm may not delete residues that are aligned with gaps if they are surrounded by high-scoring alignment positions (these residues are named GAP and are numbered using insertion codes in the generated model) and may discard residues surrounded by numerous gap positions (irrespective of whether they are aligned with residues of the target or not).

*Sculptor* also provides simple functions to polish the resulting chain trace, *e.g.* resulting short main-chain fragments can be deleted and short deletions in regular secondary structure (which possibly result from incorrect alignment) can be reinstated. Similar functionality is available in *MOLREP*, which imposes secondary-structure constraints onto the alignment, but in *Sculptor* these corrections are made by analysing the resulting trace. Similar results can be obtained by using a medium-to-long averaging window, as the smoothing provided by averaging seems to be sufficient to correct for these errors.

### 2.4. Side-chain pruning   

Sequence similarity can also be employed in determining whether or not a side chain should be truncated. *Sculptor* supports truncations to two separate levels, namely to the C^β^ atom and also to the C^γ^ atoms based on the value of the sequence similarity. Firstly, equivalent side-chain atoms are mapped (and optionally renamed) from the residue type found in the model to that in the target sequence using a simple two-dimensional graph-matching procedure. Atoms that have no counterpart are deleted (Lebedev *et al.*, 2008 ▶). The side chain is then truncated to C^β^ if the sequence similarity is below the C^γ^ threshold, truncated to C^γ^ if the sequence similarity is between the C^γ^ threshold and the full-length threshold and not truncated otherwise.

The positions of missing side-chain atoms that are determined by local geometry can be calculated and these atoms can be added to the structure. Currently, this is implemented for missing C^β^ atoms if the necessary main-chain atoms are all present. Coordinate prediction is performed by superimposing an ideal Ala on the current residue.

### 2.5. Sequence-similarity and accessible surface area-based *B*-­factor calculation   

Coordinate errors of atoms can be reflected in the model by replacing atomic *B* factors with a measure proportional to the expected error. Potential measures that are available in *Sculptor* include sequence-similarity and accessible surface-area scores. Since the proportionality constant should be negative for sequence similarity (one would expect highly homologous segments to be more conserved and therefore to have a higher weight, which corresponds to a lower *B* factor), *B* factors calculated with a linear combination could be negative. This is avoided by adding a constant if necessary so that the minimum *B* value is 10 Å^2^. In *Phaser* (McCoy *et al.*, 2007 ▶), calculated structure factors are normalized so that the results are not affected by a constant shift in *B* factors.

### 2.6. Benchmarking   

**Figure 2:**
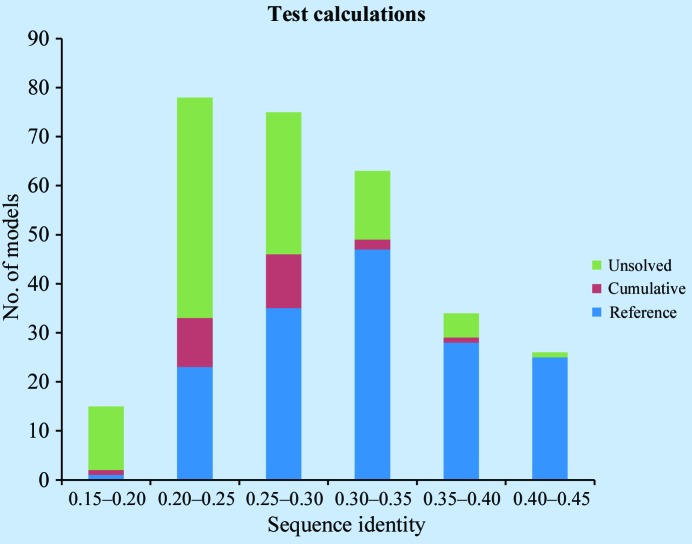
Sequence-identity distribution of models used in the benchmark suite. Solution statistics are indicated for *ClustalW* alignments. Reference, solved by the reference protocol (protocol 1); cumulative, solved by any protocols; unsolved, no solutions found.

A benchmark suite was compiled using randomly selected structures with experimental data available from the PDB (Berman *et al.*, 2002 ▶). Only cases with one molecule in the asymmetric unit were considered. For diversity, each target structure was chosen from a different SCOP (Murzin *et al.*, 1995 ▶) protein family in one of the most common SCOP protein classes (α, β, α/β and α+β). Suitable models for these targets were selected from a *BLAST* search (Altschul *et al.*, 1990 ▶). Typically, only models below 40% sequence identity were kept, although a few higher sequence-identity models were also included for particular tests. A total of 23 target structures were selected, with a total of 291 possible models (Fig. 2 ▶). Statistics of the benchmark suite are shown in Table 1 ▶.

*Sculptor* was then used to modify the models according to several protocols (Table 2 ▶). Molecular replacement was performed using *Phaser* running in automated mode and using diffraction data to 2.5 Å resolution. Map correlation coefficients (MapCC) against the target structure were calculated for potential solutions using utilities in *PHENIX* (Adams *et al.*, 2010 ▶) and molecular replacement was deemed to be successful if the MapCC was above 0.2. The solution was deemed to be identifiable if the corresponding translation-function *Z* score was above 7.0. The quality of solutions was measured in terms of their log-likelihood gain. These values were compared with the log-likelihood gains yielded by a reference protocol that corresponds to the algorithm in Schwarzenbacher *et al.* (2004 ▶) (protocol 1). Differences were normalized and averaged to a single quality parameter for a protocol. Cases were only included in the final average if both the reference and the protocol being evaluated managed to find a solution. Results are summarized in Table 3 ▶.

### 2.7. Determination of parameter values   

A preliminary study suite containing four target structures was selected from the benchmark suite (one from each SCOP class) and used to explore the effect of algorithm parameters on the quality of the resulting models. The parameters were explored on one-dimensional or two-dimensional grids that were made sufficiently large to see all effects.

In order to separate changes in model quality from other aspects of the search that may influence whether or not a solution is found, instead of performing molecular replacement the log-likelihood gain was calculated by substituting the model for a previously established solution and performing rigid-body refinement. The *Z* score was then calculated from a random sample of 500 translations.

Parameter combinations for models yielding high log-likelihood gain scores were collected and analysed to see whether correlations could be found between the score and model or alignment properties. Optimal parameter combinations and established relationships were used in defining the protocols investigated in benchmark calculations.

### 2.8. Effect of alignment accuracy   

It has been established by Schwarzenbacher *et al.* (2004 ▶) that a correct alignment is very important for optimal modification. However, since different modification algorithms may vary in their tolerance of alignment inaccuracies, comparisons were made using alignments generated as follows.

(i) A structural alignment from *LSQMAN* (Madsen & Kleywegt, 2002 ▶) was taken as the best alignment possible (macro taken from the OMAC repository). These alignments were prepared using strict spatial tolerances (3.5 Å) to ensure that residues are only put in equivalent positions if they are spatially close when superposed, so that one would model the other reasonably well.
(ii) A similar structural alignment was prepared with *LSQMAN* but with more generous spatial tolerances (8.0 Å). This results in less fragmented alignments in which moderate structural deviations (*e.g.* a loop in a different conformation) are permitted. This can be regarded as the best alignment that might be possible using an ideal sequence-alignment tool without any structural information.
(iii) *ClustalW* (Larkin *et al.*, 2007 ▶) sequence-based alignments were used to explore the effect of common alignment errors. For certain models, *FFAS* (Jaroszewski *et al.*, 2005 ▶) alignments were also used for comparison with other alignments.

## 3. Results   

Sequence-similarity-based model-editing algorithms can be regarded as generalized versions of the algorithm introduced by Schwarzenbacher *et al.* (2004 ▶). In the original algorithm, residues in the model that are aligned with residues in the target sequence are kept, whereas those that align with a gap are deleted. This can be thought of as using a simple residue–gap binary-choice matrix that gives a score of 1.0 if the residue is aligned with a residue and of −1.0 if it is aligned with a gap. This concept can be extended by using residue-substitution matrices to measure the distance between amino-acid substitutions, since certain amino-acid substitutions (*e.g.* Tyr to Phe) are less disruptive to the main-chain conformation than others. Moreover, in the case of amino-acid substitutions involving highly homologous side chains one can also expect the positions of side-chain atoms to be fairly conserved, while in the case of other substitutions this approximation has only proved to be valid up to the C^γ^ atom. These differences can be captured with an appropriate scoring matrix.

Sequence-based alignments also tend to contain smaller errors in the form of misalignments. This may have a negative effect on the resulting molecular-replacement model. One way of handling the problem is to decrease the ‘resolution’ of the alignment and spread information contained in neighbouring positions. On one hand, this averaging procedure models the effect the substitution has on the structure, namely it perturbs neighbouring positions. On the other hand, this may act as a noise filter that converts the inherently discrete nature of sequence alignments to a smooth function, reduces large variations and identifies longer-range tendencies.

### 3.1. Use of sequence similarity in model editing   

Structural differences between homologous proteins include insertions, deletions and conformational changes. It was investigated whether sequence similarity could be used to locate and correct these. A high-precision structure-based alignment was prepared; sequence-similarity values were calculated and mapped onto the main chain. This calculation was repeated using a sequence-based alignment and differences between the two results were compared visually.

**Figure 3:**
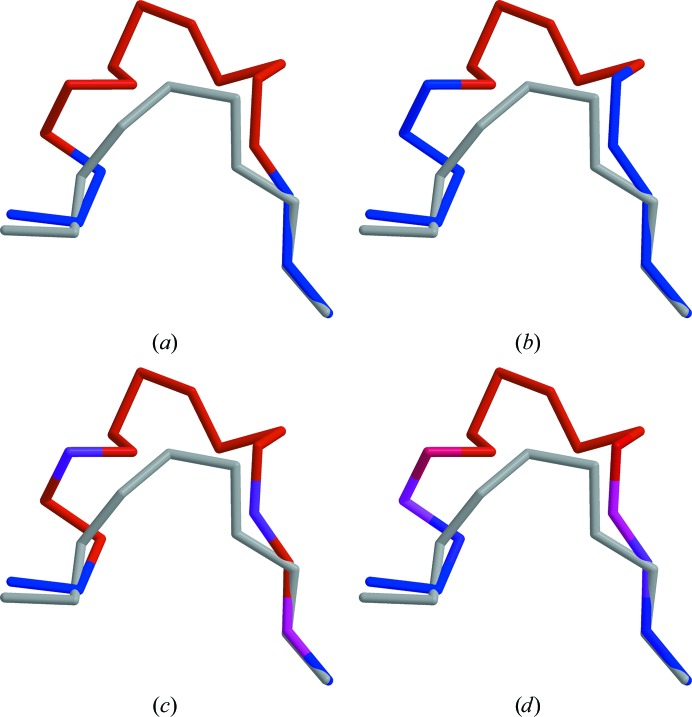
Sequence-similarity scores calculated with various settings for an insertion in the model (PDB entry 2b9l; Piao *et al.*, 2005 ▶). The target structure (PDB entry 1hj9; Leiros *et al.*, 2001 ▶) is shown in grey. Sequence similarity was calculated using (*a*) a high-precision structural alignment (target, HCY––––––––––KSGIQVR; model, HCVNSYQSNLDA­I––––KIR) using the binary scoring matrix and a null averaging window, or an *FFAS* alignment (target, HCYKS––––––GIQVR; model, HCV­NSYQSNLDAIKIR) and (*b*) the binary matrix and a null averaging window, (*c*) BLOSUM62 and a null averaging window or (*d*) BLOSUM62 and an averaging window of five. Blue indicates high sequence similarity, while red indicates areas in which sequence similarity is low. The figures were generated using *MOLSCRIPT* (Kraulis, 1991 ▶) and were rendered with *RASTER*3*D* (Merritt & Bacon, 1997 ▶).

It was found that long insertions corresponding to extra domains were easily identified from sequence-similarity scores using both alignments. The results differed more with shorter (several residue) insertions, but results from sequence-based alignments still matched those from structural alignments relatively well. A typical scenario is shown in Fig. 3 ▶. When using an averaging window of zero and the binary scoring matrix, a structural alignment clearly highlights residues that deviate spatially (Fig. 3 ▶ *a*). When using a sequence-based alignment, good results can be achieved with the binary scoring matrix and slightly better results with BLOSUM62 (Figs. 3 ▶ *b* and 3 ▶ *c*). With both matrices, the accuracy of the results can be further improved if sequence-similarity scores are averaged over neighbouring positions (Fig. 3 ▶ *d*).

**Figure 4:**
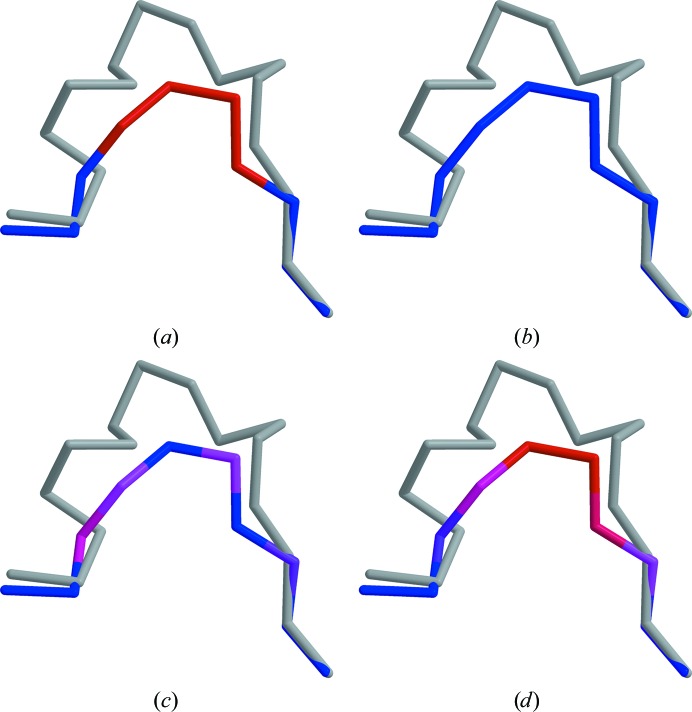
Sequence-similarity scores calculated with various settings for a deletion in the model (PDB entry 1hj9). The target structure (PDB entry 2b9l) is shown in grey. Alignments are identical to those used for Fig. 3 ▶, but the roles of target and model are reversed. Sequence similarity was calculated using (*a*) a high-precision structural alignment using the binary scoring matrix and a null averaging window, or an *FFAS* alignment and (*b*) the binary matrix with a null averaging window, (*c*) BLOSUM62 and a null averaging window or (*d*) BLOSUM62 and an averaging window of five. Blue indicates high sequence similarity, while red indicates areas in which sequence similarity is low. The figures were generated using *MOLSCRIPT* (Kraulis, 1991 ▶) and rendered with *RASTER*3*D* (Merritt & Bacon, 1997 ▶).

Interestingly, more significant differences were found when deletions were studied. Structural alignments still clearly identify deviating residues (Fig. 4 ▶ *a*). However, with an averaging window of zero no substitution matrices are able to identify the same regions (Figs. 4 ▶ *b* and 4 ▶ *c*). This asymmetry is caused by the lower precision of non-structure-based alignment algorithms, which are able to identify the presence of deletions but not conformational changes in nearby residues that result from the deletion. In this case, information about the proximity of deletions needs to be propagated to neighbouring residues. This can be achieved using longer averaging windows and the resulting sequence-similarity scores can indicate the deletions more accurately (Fig. 4 ▶ *d*).

Structural alignments also clearly indicate regions that deviate structurally. In favourable cases, structural deviations can also be detected using sequence-similarity scores calculated from sequence-based alignments. In this case, it is essential to use longer averaging windows in sequence-similarity calculations and the results are also significantly less accurate than those that could be calculated from structural alignments if structural alignments were available prior to structure solution.

### 3.2. Main-chain deletion   

To employ sequence-similarity scores for deleting residues that are not present or deviate significantly from their counterparts in the target, a suitable threshold value needs to be selected. This value was determined by performing calculations on a small sample and adjusting thresholds to optimize the log-likelihood gains of the models obtained. The presence of a correlation could be established between these quantities. It was found that optimum threshold values yielded models that contained as many residues (±5%) as there were aligned positions in the sequence alignment. It is interesting to note that the algorithm published by Schwarzenbacher *et al.* (2004 ▶) also arrives at the same number by simply deleting all residues from the model that align with gaps in the target. Several exceptions were found when *ClustalW* alignments were used, but these could all be attributed to alignment errors. Optimal models for these cases contained fewer residues than aligned positions in the sequence alignment. On the other hand, optimal values for the averaging window parameter used in sequence-similarity calculation varied unpredictably. Optimal values are potentially dependent on the spread of structural perturbations through the main chain, which is dependent on the individual case. Interestingly, the log-likelihood gains of the resulting models were not greatly affected by averaging window choice if correct deletion thresholds were used.

Three protocols were formulated and evaluated with the benchmark suite to assess performance on a large sample. In all three cases the identity matrix (1.0 if the residues are identical, −1.0 if one of them is a gap position and 0.0 otherwise) was used to calculate sequence similarity. For protocol 1, which corresponds to the algorithm in Schwarzenbacher *et al.* (2004 ▶), both the averaging window and the deletion threshold were set to zero. In protocol 2, the averaging window parameter was set to three and the threshold to a fixed value corresponding to the average of best parameter values as determined in the preliminary study over the studied sequence-identity range. In protocol 3 the averaging window parameter was set to five and the deletion threshold was chosen to give a model that contained the number of residues aligned in the corresponding sequence alignment.

Results indicate that when strict alignments from *LSQMAN* are employed a shorter averaging window is more advantageous; models generated by protocol 1 have on average 2–4% higher log-likelihood gain than those generated by protocols 3 and 2, respectively. This may be attributed to the alignment being so accurate that smearing out the signal is counterproductive. With less accurate (but more realistic) alignments (including that from *LSQMAN* with more tolerant settings), the differences are negligible. As expected from preliminary results, protocol 2 does not perform as well as protocol 3. On the other hand, all protocols occasionally lead to solutions that are not located by other protocols.

### 3.3. Side-chain pruning   

To enable the use of sequence-similarity scores in side-chain pruning similar to the algorithm of Schwarzenbacher *et al.* (2004 ▶), suitable threshold values need to be found (only single-level pruning to the C^γ^ atom was performed in order to enable comparisons with the original algorithm). These were determined from a small-scale study using the identity and BLOSUM62 matrices. The quality of the best models yielded by the algorithm with the two different scoring matrices showed only minor differences. However, short averaging windows gave better results in sequence-similarity calculations with BLOSUM62, while on the whole longer averaging windows were better with the identity matrix. The optimal pruning threshold was identical for both matrices.

To assess the performance on a larger benchmark, two protocols were formulated. Protocols 1 and 3 were modified so that side-chain pruning is performed using sequence-similarity scores calculated with the BLOSUM62 matrix, using an averaging window of one and the optimal pruning threshold (protocols 4 and 5). These protocols led to solutions with a slightly higher log-likelihood gain than those provided by protocol 1 when accurate alignments from *LSQMAN* were used (compared with results obtained with the respective basis protocols), but became progressively worse with decreasing alignment accuracy.

### 3.4. *B*-factor calculation   

#### 3.4.1. Sequence similarity   

It has been established that low values of sequence similarity correlate with large structural differences. This correlation could be exploited to weight the model structure according to expected coordinate errors. This was studied by replacing *B* factors with those calculated from sequence-similarity scores. It was established from calculations on a small sample that longer averaging windows were needed in sequence-similarity calculations for this to work well. Suitable values of the proportionality factor between sequence-similarity scores and *B* factors were also determined from this sample.

Two protocols were set up to test this method with models from the benchmark suite. As before, protocols 1 and 3 were modified to include main-chain weighting using sequence-similarity scores calculated with the BLOSUM62 matrix and an averaging window of five. To calculate atomic *B* factors from the scores, a proportionality constant of −80 (protocols 6 and 7) was used. The results indicate a strong dependence on alignment precision. The average log-likelihood gain increases by around 3% with respect to the corresponding basis protocol for structure-based alignments, but decreases by about 0.5% for alignments created with *ClustalW*. However, since sequence similarity is a property of alignment position only one *B* factor per residue could be used, which is a relatively simple *B*-factor model and may not be optimal.

#### 3.4.2. Accessible surface area   

It has been reported by Lebedev *et al.* (2008 ▶) that accessible surface area can be used to improve model quality. However, since in *Sculptor* the accessible surface area is calculated using the original structure, while in *MOLREP* this is performed after main-chain deletion, it was expected that optimal values for the proportionality factor between accessible surface area and *B* factors would differ between the two programs. The optimum for *Sculptor* was determined from calculations performed on a small sample.

Two protocols were formulated to study the method on a larger sample. As before, protocols 1 and 3 were modified to reset atomic *B* factors to values calculated from accessible surface-area values with a proportionality factor of 12 (protocols 8 and 9). Generally good results were achieved. When compared with the respective protocols that they were based on, both protocols 8 and 9 resulted in a 3–5% increase in log-likelihood gain. Interestingly, greater improvements were found with less precise alignments. This may be attributed to the frequent occurrence of incorrectly aligned residues on the surface.

It should be noted that inclusion of this procedure may lead to a worse model than the original in some circumstances (*e.g.* multimer models) and because of the selection criteria applied for the benchmark set the results may be biased. In difficult cases it is therefore worthwhile to try models generated with and without this step as well.

#### 3.4.3. Combination of accessible surface area and sequence similarity   

Accessible surface area is an independent measure from sequence similarity (although the two will be correlated) and it was studied whether a combination of the two would be more powerful. Firstly, optimal values for proportionality factors were determined for the combination (sequence similarity was calculated using the BLOSUM62 matrix with an averaging window of five). The obtained proportionality factors were similar to, although slightly lower than, the optimal values when the methods were used in isolation.

To establish the performance of the combined method, protocols 1 and 3 were modified to replace atomic *B* factors with values calculated using a combined protocol (protocols 10 and 11) and calculations were performed on the benchmark suite. Unexpectedly good results were obtained in terms of the number of solutions, number of identifiable solutions and log-likelihood gain. The increase in log-likelihood gain was about 8–9% when compared with the respective basis protocol, which is approximately twice as much as one would expect from adding up the gains achieved by the individual methods. It is likely that the combination is more tolerant to exact parameter choices and will perform optimally over a wider parameter range, since the two methods generate *B*-factor distributions that are similar and therefore one can compensate for errors in the other.

It should be noted that accessible surface-area values as calculated in *MOLREP* may include an indirect contribution from sequence similarity, since accessible surface area will increase in low-sequence-similarity regions owing to many atoms being deleted from the model.

### 3.5. All-methods combination   

The existence of further correlations between main-chain deletion, side-chain pruning and *B*-factor calculation was revealed when a protocol based on protocol 11 but also containing side-chain pruning as in protocols 4 or 5 (protocol 12) was formulated and calculations were performed using the full benchmark suite. Minor or no improvements were expected based on the established performances of protocols 4 and 5, but significant improvements were observed. This may indicate a synergistic effect between side-chain pruning (performed using sequence-similarity scores) and *B*-factor calculation, potentially the part that is based on accessible surface area. The exact mechanism of this is not clear, although it is likely to emerge as a consequence of pruning being an extreme form of *B*-factor modification.

### 3.6. Multi-protocol strategies   

The number of test cases that could be solved by any protocol was determined and was found to be significantly higher than the number of solutions obtained with any single protocol for all three alignments. Improvements in the number of solved cases were around 10% of the solutions found by the best protocol, while up to 20% more solutions could be identified from search statistics. This improvement is even more impressive when compared with the number of borderline cases, which is estimated by the number of cases that were not solved by all protocols: the multi-protocol strategy leads to 50–100% more borderline cases being solved.

It was found that a subset of all protocols would lead to the same number of solved cases. These were protocols 1, 6, 9, 10, 11 and 12 for strict *LSQMAN* alignments, protocols 2, 6, 7, 8 and 11 or 2, 6, 7, 10 and 11 for tolerant *LSQMAN* alignments and protocols 2, 6, 7, 10, 11 and 12 or 2, 7, 5, 10, 11 and 12 for *ClustalW*. There are several common features in these combinations, as follows.

(i) All combinations contain at least one protocol with a short averaging window and one protocol with a long averaging window. Protocols with a short averaging window tend to perform better with a more accurate alignment, indicated by their share dropping from 3/6 with strict *LSQMAN* alignments to 2/5 with tolerant *LSQMAN* alignments to 1/6–2/6 with *ClustalW* alignments.
(ii) There are usually several *B*-factor models used. In almost all combinations, identical models (in terms of atoms contained) are tested with three different *B*-factor protocols.
(iii) In every combination, there is at least one protocol that is among the best performers in terms of the average log-likelihood gain of models. However, certain protocols also appear in many combinations despite their seemingly low performance (*e.g.* protocols 6 and 7).

## 4. Discussion   

There are several quality indicators for the success of molecular replacement. However, when using *Phaser* the single value that has so far been found to be the most informative is the *Z* score for the translation search for the last component, for which a value above 7.0 (in the absence of certain data pathologies, *e.g.* translational pseudosymmetry) usually indicates a correct solution. On the other hand, low values of the *Z* score do not exclude success and typically one has to make a judgement taking several parameters into account, such as the number of distinct solutions with similar quality and the absolute value of the log-likelihood gain. It frequently happens that a solution has been found but it is not possible to identify it from the search statistics. In such a case, automated rebuilding calculations may identify one of the candidates as a true solution (Keegan & Winn, 2007 ▶; Long *et al.*, 2008 ▶; Schwarzenbacher *et al.*, 2008 ▶). Visual inspection of the model in the electron density is rarely helpful, as nonsolutions can also give very clear (but incorrect) maps. However, it can be useful to look for features in the map that are missing from the model.

Unfortunately, the translation-function *Z* score is dependent on many factors and is not an absolute measure of model quality. However, the log-likelihood gain is a semi-absolute quantity that can be compared between models if calculations were made using the same reflection data. There were also very strong correlations (>0.9) observed between the *Z* score and the log-likelihood gain in preliminary studies; notable exceptions were models that contained large incorrect segments owing to alignment errors. We could therefore assume that models that yield higher log-likelihood gains will also give better *Z* scores and would be more suitable for molecular replacement.

Analysis of Table 2 ▶ shows that there is generally a weak correlation between fractional log-likelihood gain and number of solutions found (0.78, 0.50 and 0.13 for strict and tolerant *LSQMAN* and *ClustalW*, respectively). On the other hand, there is a relatively strong correlation between fractional log-likelihood gain and number of identifiable solutions (0.90, 0.72 and 0.95, respectively). Therefore, protocols that yield models with higher log-likelihood gains are more likely to provide solutions that can be identified from search statistics. Exploring a range of protocols can be instrumental in covering model space and finding unique solutions.

### 4.1. *B*-factor calculations   

There are multiple criteria that optimal *B* factors should conform to: they have to match the *B*-factor distribution of the target structure and at the same time downweight structural regions that potentially differ between the target and the model. The original *B* factors of the model are therefore a good default choice since they provide a *B*-factor distribution that resembles a real protein structure. However, they are influenced by crystal packing, which is not a transferable property from one protein model onto another. It may therefore be advantageous to replace the original *B* factors with those calculated from other sources. It was found that a combination of *B* factors calculated from accessible surface-area and sequence-similarity values performs favourably and may be used as a default choice. An alternative method would be the combination of the original *B* factors with those calculated from sequence-similarity scores, in which case the original model *B* factors would be responsible for matching the *B*-­factor distribution of the target, while those calculated from sequence similarity would downweight potentially different regions.

### 4.2. Diversity of resulting models   

By combining the results of all protocols used in benchmark runs, a significantly larger number of cases could be solved than with any single model alone. This indicates that by changing algorithm parameters or enabling/disabling modification techniques such as *B*-factor adjustment, the structures obtained are sufficiently diverse that they could be employed in molecular replacement as unique models although they are based on the same structural template. Similar behaviour can be observed on a set of ensemble models truncated to various r.m.s.d. cutoffs (Konagurthu *et al.*, 2010 ▶). Exploring all homologues first would possibly still be a more efficient approach.

### 4.3. Multisolution approach   

It is tempting to speculate on how the best coverage of accessible model space could be achieved. Based on observations made on best-performing protocol combinations, the following may be beneficial.

(i) Varying the averaging-window parameter for main-chain deletion can have a large effect on the resulting trace. Good results could be obtained when a combination of one short and one longer averaging window was used. This may be improved slightly by trying additional values. Longer averaging windows perform better for less accurate alignments.
(ii) Calculating atomic *B* factors according to simple *B*-­factor models (sequence similarity, accessible surface area) works surprisingly well. It may therefore be beneficial to explore additional *B*-factor models. One possibility would be to employ original model *B* factors to estimate the *B*-­factor distribution of the target in combination with predicted co­ordinate error weighting calculated from sequence-similarity and accessible surface-area values.

To some extent, the powerful but computationally intensive combinatorial trimming technique described by Schwarzenbacher *et al.* (2008 ▶) can be approximated by this approach. It is not clear how many different protocols are necessary to obtain optimal performance, but a moderate number seems to give good results.

### 4.4. Effect of alignment accuracy   

Benchmark results indicate that accurate alignments would enable more models to be successfully used in molecular replacement (Schwarzenbacher *et al.*, 2004 ▶). Interestingly, there even seem to be differences in success rate between high-precision *LSQMAN* and more tolerant *LSQMAN* alignments. *ClustalW* alignments are significantly less successful, although one has to note that *ClustalW* has not been optimized for the studied sequence-similarity range. Limited calculations performed using *FFAS* alignments suggest that their success rate is fairly close to that of structural alignments.

When cumulative results with models generated from a single template using several protocols are compared the differences are minute and *ClustalW* performs almost as well (at least in terms of the number of solved structures) as a structural alignment prepared using tolerant settings, which approximates the best that might be achieved by sequence-based alignments. This may indicate that smaller errors can be compensated efficiently by using a number of protocols. More accurate alignments still seem to be important in helping solutions stand out from the noise, as indicated by the number of identifiable solutions being higher for structure-based alignments.

### 4.5. Visual editing functionality   

*Sculptor* has many options and their effects on the resulting structure are somewhat obscure. Therefore, an interface between *Sculptor* and *Coot* (Emsley *et al.*, 2010 ▶) has been developed, originally to visualize the effect of algorithm parameters on main-chain and side-chain atoms. When a protocol is set up, the interface provides visual feedback indicating residues that will be deleted and makes it easy to visually optimize parameters to obtain plausible models that are not fragmented but have likely flexible parts deleted. Inability to obtain such models may indicate alignment errors. Manual main-chain editing is also supported to enable the user to make smaller modifications to the resulting trace. The interface allows quick model generation, so that a series of models can be created and tried in molecular replacement straight away.

## 5. Conclusions   

The model-editing program *Sculptor* has been written to prepare and correct models for molecular replacement and offers a selection of editing algorithms with a flexible interface to define processing protocols. Implementing several algorithms in one program has the additional benefit that available algorithms can also be used in combination, increasing the number of possibilities even further. In addition, novel algorithms have been developed that can utilize the information contained in sequence alignments (including multiple sequence alignments) to a larger extent. The combination of all these features makes *Sculptor* a powerful tool. A visual interface (utilizing the *Coot* toolkit) has also been developed to help users experiment with available options and define optimal protocols.

Benchmarks performed with *Sculptor* confirm that it can generate a diverse set of molecular-replacement models from a single template structure. Although some of these models may perform better than those that are in current use, significantly better results can be obtained if these are employed as alternatives in a molecular-replacement search. This strategy can increase the success rate by up to 30% in the 20–30% sequence-identity region and can also compensate for alignment inaccuracies. A selection of protocols that maximizes diversity would therefore enhance the success rate in molecular-replacement pipelines.

*Sculptor* (including the *Sculptor–Coot* interface) is currently available in the *PHENIX* suite (http://www.phenix-online.org). Work is ongoing on incorporating it into the *CCP*4 suite and the development of a *ccp*4*i* GUI is also in progress.

## Figures and Tables

**Table 1:** Statistics of the benchmark suite Targets marked with asterisks were also included in the preliminary study suite, with the number of models shown in parentheses.

Target	SCOP family	No. of residues	Data resolution ()	No. of models	Sequence-identity range (%)
1aa2	a.40.1.1	108	2.01	8	21.330.6
1az5	b.50.1.1	99	2.00	14	20.236.4
1cm3	d.94.1.1	85	1.60	12	18.838.8
1emf	d.22.1.1	225	2.40	13	20.937.1
1hj9* (15)	b.47.1.2	230	0.95	15	24.240.8
1hp7	e.1.1.1	376	2.10	20	21.847.1
1iom	a.103.1.1	374	1.50	11	25.247.7
1jdl* (10)	a.3.1.1	118	1.70	16	19.038.0
1lds	b.1.1.2	97	1.80	19	18.046.5
1n9n	d.110.3.6	108	2.30	13	15.637.6
1npl	b.78.1.1	109	2.00	5	16.528.4
1o4v	c.23.8.1	183	1.77	3	15.321.3
1phw	c.1.10.4	284	2.36	6	20.627.9
1r29* (10)	d.42.1.1	127	1.30	14	20.538.7
1upi	b.82.1.1	225	1.70	14	25.340.5
1xcd	c.10.2.7	329	2.31	11	19.832.7
1z45	b.30.5.4 + c.2.1.2	699	1.85	19	20.954.0
1zzo	c.47.1.10	136	1.60	14	17.627.9
2azz	a.133.1.2	124	2.20	19	18.245.9
2dhq	c.23.13.1	146	2.00	8	20.536.3
2i5f	b.55.1.1	109	1.35	14	17.435.8
2iyw* (5)	c.37.1.2	184	1.85	12	16.337.0
1nqc	b.1.18.10	138	2.05	11	22.538.3

**Table 2:** Protocols used in benchmark calculations For main-chain deletion, only the identity scoring matrix was employed. ‘Variable’ thresholds were calculated from the respective sequence alignment. Protocol 1 corresponds to the algorithm published by Schwarzenbacher *et al.* (2004 ▶).

Protocol	Main-chain window	Threshold	Side-chain matrix	Window	Threshold	*B* factor
1	0	0.0	Identity	0	1.0	Original
2	3	0.2	Identity	0	1.0	Original
3	5	Variable	Identity	0	1.0	Original
4	0	0.0	BLOSUM62	1	0.2	Original
5	5	Variable	BLOSUM62	1	0.2	Original
6	0	0.0	Identity	0	1.0	Sequence-similarity based (matrix = BLOSUM62, window = 5, factor = 80)
7	5	Variable	Identity	0	1.0	Sequence-similarity based (matrix = BLOSUM62, window = 5, factor = 80)
8	0	0.0	Identity	0	1.0	Accessible surface area-based (factor = 12)
9	5	Variable	Identity	0	1.0	Accessible surface area-based (factor = 12)
10	0	0.0	Identity	0	1.0	Sequence-similarity based (matrix = BLOSUM62, window = 5, factor = 60) + accessible surface area-based (factor = 8)
11	5	Variable	Identity	0	1.0	Sequence-similarity based (matrix = BLOSUM62, window = 5, factor = 60) + accessible surface area-based (factor = 8)
12	5	Variable	BLOSUM62	1	0.2	Sequence-similarity based (matrix = BLOSUM62, window = 5, factor = 60) + accessible surface area-based (factor = 8)

**Table 3:** Comparison of results from benchmark jobs There are a total of 291 models in the benchmark suite. Protocol descriptions are shown in Table 2 ▶. Any, solved by any protocols; All, solved by all protocols. Solved, a solution with MapCC 0.2 (with respect to the target structure) has been found. Identifiable, a solution with TFZ 7.0 has been found. Gain: average fractional difference in log-likelihood gain with respect to protocol 1.

	*LSQMAN* (strict)			*LSQMAN* (tolerant)			*ClustalW*		
Protocol	Solved	Identifiable	Gain	Solved	Identifiable	Gain	Solved	Identifiable	Gain
1	183	126		173	119		159	102	
2	182	127	0.042	174	115	0.003	163	104	0.001
3	182	124	0.021	173	112	0.002	165	102	0.005
4	178	127	0.004	179	123	0.007	160	99	0.011
5	182	126	0.018	173	116	0.007	160	101	0.012
6	185	129	0.035	182	127	0.026	164	100	0.007
7	182	129	0.006	181	118	0.016	162	103	0.002
8	182	134	0.033	177	123	0.031	158	108	0.050
9	185	129	0.009	173	122	0.028	165	109	0.056
10	188	135	0.093	180	128	0.096	162	113	0.098
11	186	133	0.066	181	130	0.089	162	113	0.100
12	188	136	0.092	178	124	0.114	163	110	0.113
Any	199	146		190	137		183	123	
All	165	111		155	99		134	87	
